# Metadata recommendations for light logging and dosimetry datasets

**DOI:** 10.1186/s44247-024-00113-9

**Published:** 2024-08-28

**Authors:** Manuel Spitschan, Grégory Hammad, Christine Blume, Christina Schmidt, Debra J. Skene, Katharina Wulff, Nayantara Santhi, Johannes Zauner, Mirjam Münch

**Affiliations:** 1 Translational Sensory & Circadian Neuroscience, Max Planck Institute for Biological Cybernetics, Tübingen, Germany; 2 Chronobiology & Health, TUM School of Medicine and Health, Technical University of Munich, Munich, Germany; 3 TUM Institute for Advanced Study (TUM-IAS), Technical University of Munich, Garching, Germany; 4 Chronobiology, Faculty of Health and Medical Sciences, University of Surrey, Guildford, UK; 5 Sleep & Chronobiology Group, GIGA-CRC-In Vivo Imaging Research Unit, University of Liège, Liège, Belgium; 6 Chair of Neurogenetics, Institute of Human Genetics, University Hospital, Technical University of Munich, Munich, Germany; 7 Centre for Chronobiology, Psychiatric Hospital of the University of Basel, Basel, Switzerland; 8 Research Cluster Molecular and Cognitive Neurosciences, University of Basel, Basel, Switzerland; 9 Department of Biomedicine, University of Basel, Basel, Switzerland; 10 Psychology and Neuroscience of Cognition Research Unit (PsyNCog), Faculty of Psychology, Speech and Language, University of Liège, Liège, Belgium; 11 Department of Molecular Biology, Umeå University, Umeå, Sweden; 12 Wallenberg Centre for Molecular Medicine (WCMM), Umeå University, Umeå, Sweden; 13 Department of Psychology, Northumbria University, Newcastle, UK

**Keywords:** Personal light exposure, Light logging, Light logger, Metadata, Json, Metadata descriptor, Non-visual effects of light, Melanopic, Melanopsin, Iprgc

## Abstract

**Background:**

Light exposure significantly impacts human health, regulating our circadian clock, sleep–wake cycle and other physiological processes. With the emergence of wearable light loggers and dosimeters, research on real-world light exposure effects is growing. There is a critical need to standardize data collection and documentation across studies.

**Results:**

This article proposes a new metadata descriptor designed to capture crucial information within personalized light exposure datasets collected with wearable light loggers and dosimeters. The descriptor, developed collaboratively by international experts, has a modular structure for future expansion and customization. It covers four key domains: study design, participant characteristics, dataset details, and device specifications. Each domain includes specific metadata fields for comprehensive documentation. The user-friendly descriptor is available in JSON format. A web interface simplifies generating compliant JSON files for broad accessibility. Version control allows for future improvements.

**Conclusions:**

Our metadata descriptor empowers researchers to enhance the quality and value of their light dosimetry datasets by making them FAIR (findable, accessible, interoperable and reusable). Ultimately, its adoption will advance our understanding of how light exposure affects human physiology and behaviour in real-world settings.

## Introduction

In this article, we are proposing a novel metadata descriptor for obtaining key metadata information in personalized light exposure data sets. Metadata holds information about many elements in a dataset, e.g. location coordinates, exposure duration and the individual circumstances in which it was collected, all of which provide context for meaningful analysis. Light has a fundamental impact on human physiology and behaviour, beyond vision [1–3]. It serves as the primary zeitgeber or ‘time signal’ for the human circadian system, allowing it to synchronise physiological and behavioural functions to the external light–dark cycle. In addition to its synchronising effect, light exposure can also modulate melatonin [4–7], alertness [8–10] and cognitive performance [11], and influence sleep architecture [12], thermoregulation and the cardiovascular system [13]. Light receptors in the eye, especially the melanopsin-containing retinal ganglion cells, with their peak sensitivity at the blue end of the light spectrum, play a dominant role in this. Thus, the physiological and behavioural influences of light are subsumed under the heading “non-visual” or melanopic effects of light, demarcating them from the visual effects of light, e.g. seeing and perceiving motion, colour and space in the world.

While most mechanistic insights on the non-visual effects of light come from controlled laboratory studies with exposure to constant or parametric modulations of artificial light, there is now an emerging literature on the impact of “real-world” light exposure under ambulatory, daily life conditions [14]. In these studies, participants are usually given wearable light dosimeters which capture light exposure over several hours, days or even weeks. These light dosimeters can be placed at different locations, including the wrist using a watch-like wristband, on the chest as a brooch or pendant, or attached to spectacle frames in the direction of gaze [14]. Additionally, they have different optical properties and performance characteristics [15–17]. Especially wrist-worn devices, which often primarily measure activity using accelerometers, now also include different types of light sensors. However, many of them do not estimate melanopic effects (i.e., effects on melanopsin-containing intrinsically photosensitive retinal ganglion cells [ipRGCs]) of light and thus fail to predict its circadian impact. More recently, light dosimeters have been developed that also incorporate the short-wavelength spectral sensitivity of melanopsin [18–20]. Individual light exposure patterns from such sensors have further been included in mathematical models to predict parameters of circadian physiology [21, 22].

The exact light exposure that an individual receives over time depends on a range of factors [23, 24]. At the individual level, light exposure depends on occupation [25–27], age [28, 29], chronotype, and health status [28, 30–32]. Additionally, geographical and seasonal variations in photoperiod length [33–38] and illuminance levels give rise to differences in available daylight [39, 40]. Given this variability in individual light exposure patterns, there is a need to combine datasets collected in different cohorts across different socio-economic, seasonal, and geographical contexts.

To ensure that data collected by different research groups are comparable and can be combined where needed, it is essential to document the conditions which have generated these data. These metadata, i.e., data about the data, have to record which device was used, the context in which it was generated and the descriptors of the participant. More broadly, metadata are key to make data findable, accessible, interoperable and reusable (FAIR, [41]), and seen key as components to support data sharing mandates from funders, journals and institutions [42, 43]. Over the last decades, infrastructure has been established for sharing data, with generalist platforms such as Zenodo (https://zenodo.org/), FigShare (https://figshare.org/) or the Open Science Framework (https://osf.io/). Within different areas of biomedical research, specialized metadata descriptors have been developed (e.g., [44–48]). Furthermore, there is an active scholarly community working specifically on theory and practice of metadata [49–53]. The importance of standardization and metadata are emerging to be recognized in the domain of sleep and circadian science [53–56], including the establishment of the US-based NIH-funded National Sleep Research Resource [57] which also provides bespoke tooling to access and process their data [58, 59]. At present, there is no personalized metadata schema for light logging and dosimetry.

Here, we propose a metadata descriptor for light dosimetry data, incorporating study-level, participant-level, dataset-level and device-level metadata. The motivation for creating a metadata descriptor for light logging and dosimetry data stems from the need to standardize and enhance research in the field of light-related studies. This descriptor enables researchers to systematically document essential information about light exposure data, promoting reproducibility and comparability across studies. One key benefit is its facilitation of meta-analysis, allowing for comprehensive data synthesis and more robust conclusions. Additionally, it improves the overall quality and transparency of research, aiding peer review and interdisciplinary collaboration, as insights from lighting research intersect with various fields. Finally, journals, funders and institutions may also require the storage and sharing of data in a harmonized way.

## Methods

### Development of metadata descriptor

The metadata descriptor was developed by an international team of authors, from diverse scientific backgrounds (sleep research, chronobiology, vision science, psychology, neuroscience, lighting science, physics, computer science) with experience in complex, real-world data collection, through a joint development process. A series of synchronous Zoom-based discussions were held between 2020 and 2021. After an initial scoping survey and brainstorming discussions, the authors developed different thematic domains to be featured in the metadata descriptor and filled with specific items. The descriptor was refined through an iterative process using feedback given through a collaborative web-based document platform, and subsequently brought into the current final form by author M.S. This draft was then subject to a time-restricting ‘veto’ process to highlight any further disagreements. Final fine-tuning of the metadata descriptors was performed in a small-group discussion with authors J.Z., K.W., M.M. and M.S.

### Structure/hierarchy of the metadata descriptor

**Fig. 1:**
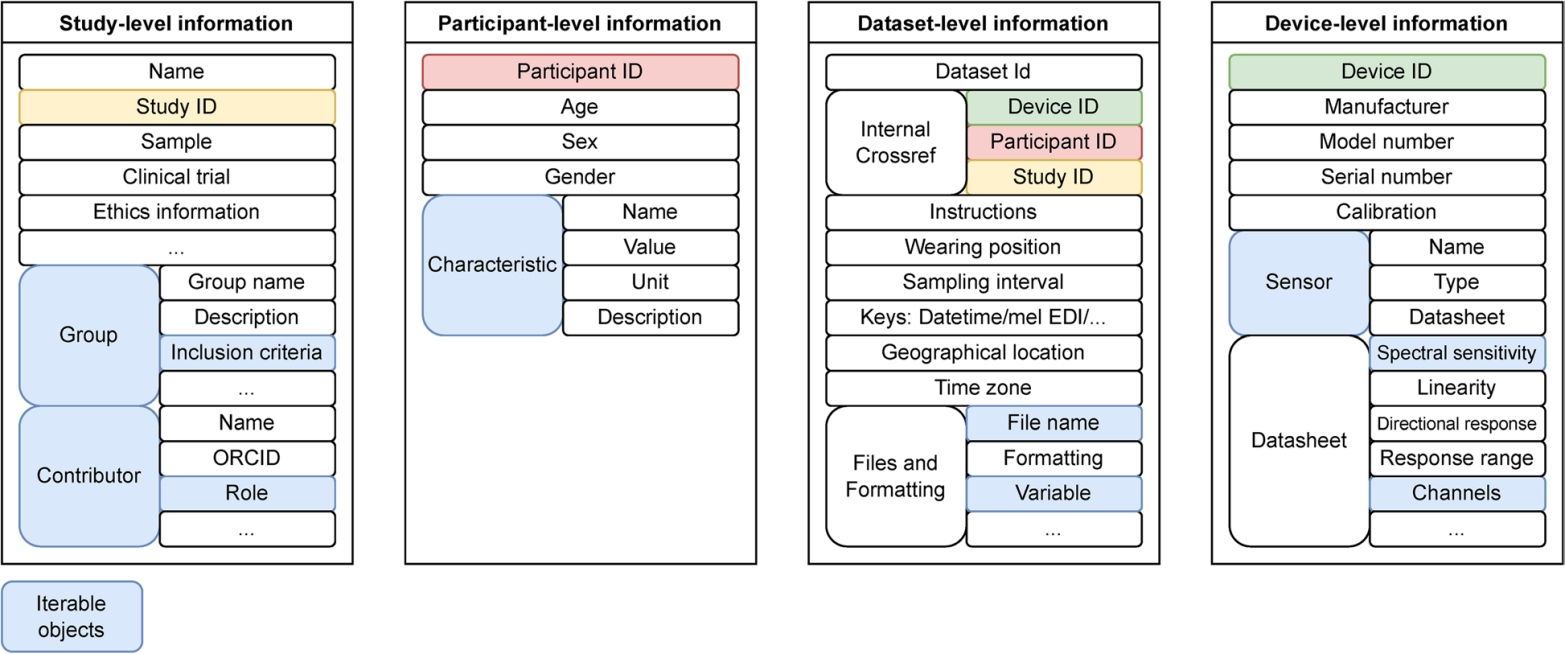
Overview of the metadata descriptor. For clarity, only first- and second-level items are shown

The metadata descriptor collects essential information across different domains of a light dosimetry dataset. This includes obligatory information about (i) the *study*, including its name, whether it is a clinical trial, a description of the study sample and different groups therein, inclusion/exclusion criteria, and contributors, (ii) the *participants*, including their age, sex and characteristics, (iii) the *dataset(s)* (at the participant level), including instructions to the participant and wear time, and (iv) the *device(s)* used, including manufacturer, model, serial number, and information about the sensors. Below, we describe in greater detail the information needed for each of these categories. The modular architecture is shown in Fig. 1. In principle, the metadata descriptor can be expanded to include additional categories.

#### Study-level information

It is important to capture metadata about a given study. Here, we consider a *study* to be a concerted data collection effort using a specific protocol. This could be a longitudinal protocol (studying a cohort over time), an observational protocol or other protocols. At the study level, we record information about the study, participant groups in the study and contributors to the study.

The study-level information includes the following items:

**Table:** 

Component	Level	Name	Required	Type	Description
Study (study.json)	0				Study-level metadata
	1	study_title	Yes		
	1	study_internal_id	Yes	string	Unique identifier for study
	1	study_preregistration	No	string	DOI (Digital Object Identifier) of pre-registration document describing data collection
	1	study_ethics	No	string	Name of ethics committee and approval number
	1	study_registration	No	string	Registry and ID of clinical trial registration
	1	study_short_description	Yes	string	Short narrative description of the study
	1	study_sample	Yes	string	Short description of the study sample
	1	study_groups		array | #/definitions/group	Groups in the study
	2	study_group		object	Group descriptor object
	3	study_group_name	Yes	string	Group name
	3	study_group_description	No	string	Group description
	3	study_group_size	No	integer	Sample size
	3	study_group_inclusion	No	array of strings (min. 0)	Inclusion criteria for sample group, given as an array of strings
	3	study_group_exclusion	No	array of strings (min. 0)	Exclusion criteria for sample group, given as an array of strings
	1	study_intervention	No	string	Short description of the study intervention, if any
	1	study_setting	Yes	string	Description of the study setting
	1	study_geographical location	Yes	string	Geographical location and context (cityrural, urban)
	1	study_contributors	No	array | contributor.json	Any contributors to the study
	1	study_datasets	Yes	array	Datasets contained within the study
	1	study_type			
	1	study_funding_sources	No	array of strings (min. 1)	Any funding sources supporting the project. If the funding number is available, it should be given”
	1	study_keywords	No	array	Key words describing the projects

At the level of contributors, the “Data curation” role (https://credit.niso.org/contributor-roles/data-curation/) must be defined. While there are key issues around data ownership that go well beyond the scope of this article, it is recommended that the research group involved in the data collection effort discusses data curation and licensing. The contributor schema is given as follows:

**Table:** 

Component	Level	Name	Required	Type	Description
Contributor (contributor.json)	0			object	Descriptor for contributor to the study
	1	contributor_full_name	Yes	string	
	1	contributor_roles	No	array of strings	
	1	contributor_email	No	idn-email	Email address
	1	contributor_orcid	Yes	string	ORCID identifier
	1	contributor_institution	No	object	Institution
	2	contributor_institution_name	Yes	string	Name of institution
	2	contributor_institution_city	No	string	City of institution
	2	contributor_institution_country	Yes	string	Country of institution

#### Participant-level information

To be able to document the type of study sample from which a light dosimetry data set was generated, it is important to include information about the participants. The participant-level information helps to identify participant characteristics, including demographics, and in particular, facilitates the merging of different datasets indexed in the database for aggregated analyses. To ensure participant anonymity, the information here should exclude personally identifiable information. To include arbitrary participant-level characteristics that were collected alongside the primary data, e.g., iris colour, handedness, or similar, we provide a reusable “Participant characteristics” metadata field.

The participant-level information contains the following items:

**Table:** 

Component	Level	Name	Required	Type	Description
Participant (participant.json)	0			object	Descriptor for study participant
	1	participant_internal_id	Yes	string	Unique ID for participant
	1	participant_age	Yes	integer	Age of the participant at the time of first participation
	1	participant_sex	No	string	Sex of participant, if recorded
	1	participant_gender	No	string	Gender of participant, if required
	1	participant_characteristic	No	array of objects	Biological and non-biological characteristics of participant
	2	participant_characteristic_name	Yes	string	Name of the characteristic
	2	participant_characteristic_value	Yes	string	Value of the charactericstic
	2	participant_characteristic_unit	No	String	Unit of the characteristic
	2	participant_characteristic_description	No	string	Description of the characteristic

#### Dataset-level information

Here, a dataset refers to an individual participant’s dataset. As it is sometimes necessary to add auxiliary data to properly analyze light measurements (such as data from a wear log) the option to add such datasets is included in the descriptor. The dataset-level information includes the following items:

**Table:** 

Component	Level	Name	Required	Type	Description
Dataset (dataset.json)	0			object	Dataset-level metadata
	1	dataset_internal_id	Yes	String	Unique identifier of dataset
	1	dataset_instructions	Yes	string	Description of the instructions that were given to the study participants before or during the collection of this data set
	1	dataset_crossref	Yes	object	Crossreferencing information
	2	dataset_crossref_study_id	Yes	string	Internal ID for study
	2	dataset_crossref_participant_id	Yes	string	Internal ID for participant
	2	dataset_crossref_device_id	Yes	string	Internal ID for device
	1	dataset_device_location	Yes	string	Anatomical location of the acquisition device
	1	dataset_sampling_interval	Yes	numeric	Sampling interval
	1	dataset_datetime	Yes	object	Name of the datetime column
	2	dataset_datetime_date	Yes	string	Name of the date column or datetime column
	2	dataset_datetime_dateformat	Yes	string	Formatting of the date column (e.g., “YYYY/MM/DD” or datetime column (e.g. “YYYY/MM/DD HH:MM:SS”)
	2	dataset_datetime_time	Yes	string	Name of the time column (only if separate from date)
	2	dataset_datetime_timeformat	Yes	string	Formatting of the time column (e.g., “HH:MM:SS”) (only if separate from date)
	1	dataset_Illuminance	Yes	string	column name in the data that contains photopic illuminance
	1	dataset_melEDI	No	string	column name in the data that contains melanopic EDI (D65)
	1	dataset_timezone	Yes	string	Timezone of data collection (Olson database)
	1	dataset_location	Yes	array of strings	Latitude/Longitude of data collection
	1	dataset_file	Yes	array of objects	Dataset descriptors
	2	dataset_file_names	Yes	array of strings	File names corresponding
	2	dataset_file_format	Yes	string	File format
	2	dataset_file_encoding	Yes	array of strings	File text encoding (e.g., UTF-8)
	2	dataset_file_timezone	Yes	string	Timezone of data (Olson database)
	2	dataset_file_auxiliary	Yes	boolean	Indicator whether the data files contain light data (or auxiliary data)
	2	dataset_file_preprocessing	Yes	object	Preprocessing Information
	3	dataset_file_preprocessing_bol	Yes	boolean	Indicator whether preprocessing was applied
	3	dataset_file_preprocessing_desc	No	array of strings	Description what preprocessing was applied (conditional requirement)
	2	dataset_file_variables	Yes	array of objects	Variables contained in the data set, units and location (column)
	3	dataset_file_variables_name	Yes	string	Variable name as contained in the dataset
	3	dataset_file_variables_labels	Yes	string	Variable name as clear name
	3	dataset_file_variables_units	Yes	string	unit
	3	dataset_file_variables_calibration	No	string	Description of transformation that should be applied to the variable for calibration. Based only on researchers’ calibration

#### Device-level information

Information about the internal workings of the data collection devices is crucial for correct analyses and outcome. Additionally, we include information about the specific sensors, such as light channels to capture information about the types of light quantities that were recorded. The motivation to use this information is to enable analyses separated by the type of device used. The device-level information contains the following items:

**Table:** 

Component	Level	Name	Required	Type	Description
Device (device.json)	0			object	
	1	device_internal_id	Yes	string	Unique ID of the device
	1	device_manufacturer	Yes	string	Manufacturer of the device
	1	device_model	Yes	string	Model of the device
	1	device_serial_number	Yes	string	Serial number of the device
	1	device_calibration	Yes	string	Date the device was last calibrated
	1	device_sensor	No	array of objects	Individual sensors
	2	device_sensor_type	Yes	string	Type of the sensor
	2	device_sensor_datasheet	No	object | device_datasheet.json	Sensor datasheet information
	1	device_datasheet	Yes	object | device_datasheet.json	Device datasheet information

At the time of publication there are efforts undertaken by the Joint Technical Committee 20 of the International Commission on Illumination (CIE) (https://cie.co.at/technicalcommittees/wearable-alpha-opic-dosimetry-and-light-logging-methods-limitations-device) and the MeLiDos project [60]. The proposed metadata descriptor uses an interface at the device level for a future descriptor specifically covering topics of accuracy and calibration, as well as standard output channels. The following table shows a cautious attempt at such a datasheet metadata descriptor for devices and sensors to showcase the possible range of such a descriptor.

**Table:** 

Component	Level	Name	Required	Type	Description
Device/Sensor Datasheet (device_datasheet.json)	0			object	
	1	datasheet_manufacturer	Yes	string	Manufacturer of the sensor/device
	1	datasheet_type	Yes	string	Type of the sensor/device
	1	datasheet_model	Yes	string	Model of the sensor/device
	1	Datasheet_calibration_interval	Yes	number	Required device calibration interval (in days)
	1	datasheet_calibration_spectral_sensitivity	Yes	Array of objects	Information about spectral sensitivity calibration
	2	datasheet_calibration_spectral_sensitivity_ wavelength	Yes	number	Wavelength (nm)
	2	datasheet_calibration_spectral_sensitivity_ relative	Yes	number	Relative spectral sensititivty at given wavelength
	1	datasheet_calibration_linearity	Yes	string	Information about linearity calibration
	1	datasheet_calibration_directional_response	Yes	string	Information about directional response calibration
	1	datasheet_calibration_range	Yes	string	Information about response range
	1	datasheet_channel	No	array of objects	Information on channels
	2	datasheet_channel_nr	Yes	integer	Number of channel
	2	datasheet_channel_name	Yes	string	Name of the channel as appearing in the export (file)
	2	datasheet_channel_unit	No	string	Unit of channel
	2	datasheet_channel_description	No	strings	Description of channel

## Discussion

### Limitations

Here, we provided the first metadata descriptor for personalized light exposure data. We wish to highlight the following limitations, for which we provide mitigating strategies under “Future directions”:

- General applicability. One limitation of the proposed metadata descriptor is its potential limited applicability to specific contexts or types of studies. While it was developed collaboratively by an international team of experts with extensive expertise in real-world data collection, certain study designs or devices may not be adequately represented or documented by the proposed metadata fields. This limitation may affect the descriptor’s ability to comprehensively capture metadata across current and future variations of light logging research. This may also include novel technologies, such as spatially resolved measurements.
- Validation and independent evaluation: We do not provide concrete evidence of validation or independent evaluation in the current paper. This lack of empirical validation may raise concerns about the descriptor’s robustness and effectiveness in different research settings. Without demonstrated validation, the community may question the reliability and accuracy of the metadata captured by the descriptor, potentially limiting its widespread adoption and acceptance. We see future opportunities to address this, including through official standards bodies.
- Challenges in implementation: While the metadata descriptor is available in JavaScript Object Notation (JSON) format and comes with a user-friendly web interface for generating compliant files, potential challenges in its implementation across various software languages and platforms are not extensively discussed. The descriptor’s compatibility with different data repositories and platforms is crucial for seamless integration into existing research infrastructures. The absence of a detailed discussion on potential implementation challenges and strategies to address them could hinder the descriptor’s practical adoption by researchers using diverse technologies and tools. A robust landscape of tooling to support different entry points will need to be developed.

### Future directions

We see the follow avenues for future work:

- Validation of the metadata descriptor in real-world settings: As we move forward, a critical step is the validation of this metadata descriptor in real-world settings across a variety of users and research contexts. This entails applying the descriptor to diverse light dosimetry datasets collected in different environments, populations, and under varying conditions, including in clinical contexts. This validation process will help ensure the descriptor’s adaptability and effectiveness in capturing the nuances of personalized light exposure data. Researchers should collaborate to assess its utility and identify potential improvements systematically.
- Independent evaluation of the metadata descriptor: To establish its robustness and credibility, independent evaluation of the metadata descriptor is imperative. Encouraging third-party assessments and peer reviews will provide valuable feedback and insights into its usability and reliability. This independent evaluation should include comparisons with existing metadata schemas and assessments of its compatibility with different data analysis tools and platforms.
- Further development through community engagement: The evolution of the metadata descriptor should involve building a collaborative community of contributors and users. Encouraging researchers, institutions, and organizations to participate in its development and maintenance actively will enhance its completeness and relevance. Continuous feedback and contributions from the community, including from device manufacturers, will be essential for keeping the descriptor up-to-date with emerging research needs and technological advancements.
- Implementing multiple entry points into the metadata descriptor: To maximize its accessibility and usability, efforts should be made to provide implementations of the metadata descriptor in multiple software languages commonly used in scientific research. In addition to the existing JSON format and web interface, providing code and tools in other languages, such as R, Python, or MATLAB, will accommodate researchers who prefer different analytical environments. This multi-language support will broaden the user base and encourage widespread adoption.
- Uptake and approval by scientific and technical organizations: A crucial future direction is to garner the uptake and official approval of the metadata descriptor by scientific and technical organizations with expertise in light exposure research and standards development. Organizations such as the Daylight Academy, which played a pivotal role in the inception of this project, and the International Commission on Illumination (CIE), the international authority in lighting and illumination standards, should be actively engaged. Collaboration with these organizations can lead to the endorsement and integration of the metadata descriptor into industry standards and guidelines, thereby enhancing its credibility and facilitating its widespread adoption within the scientific and professional community.
- Integration with data repositories and platforms: To streamline the use of the metadata descriptor, it should be integrated into existing data repositories and platforms used by researchers in the field of chronobiology and related disciplines. Creating plugins or extensions that enable seamless incorporation of metadata into data management systems will encourage researchers to adhere to the descriptor’s guidelines. This integration will not only enhance data discoverability but also simplify the process of sharing and accessing light dosimetry datasets, further promoting the FAIR principles and facilitating collaborative research efforts.

Incorporating these future directions will not only strengthen the metadata descriptor’s utility but also foster a collaborative and dynamic research community focused on advancing our understanding of the non-visual effects of light. By continuously refining and expanding the descriptor, we can collectively contribute to the FAIR principles, making light dosimetry data more accessible, interpretable, and impactful in the fields of chronobiology, sleep science, and beyond.

## Conclusion

In conclusion, the development of this metadata descriptor for light dosimetry data is a significant contribution to the field of chronobiology and personalized light exposure research. This descriptor addresses the critical need for standardized documentation of metadata associated with light exposure datasets, ensuring that data collected across various studies, contexts, and devices can be compared and utilized effectively. The modular architecture of the metadata descriptor allows for flexibility and scalability, accommodating potential future expansions.

The implementation of the metadata descriptor in JSON format, along with the user-friendly web interface for generating compliant JSON files, enhances its accessibility and usability within the research community. Furthermore, the provision of versioning ensures that the descriptor remains up-to-date and adaptable to evolving research needs.

Ultimately, this metadata descriptor facilitates the principles of FAIR data (findable, accessible, interoperable, and reusable), promoting collaboration, data sharing, and the advancement of knowledge in the study of light exposure’s effects on human physiology and behavior. Researchers and institutions are encouraged to adopt this descriptor to improve the quality and utility of their light dosimetry datasets, contributing to a more comprehensive understanding of the non-visual effects of light in real-world settings.

## Data Availability

All data and materials are available at https://github.com/tscnlab/LightExposure-MD-Schema and https://github.com/tscnlab/LightExposure-MD-Validator.
